# High-Fat Diet Provokes Aberrant Erythropoiesis in IRP1-Deleted Mice, Leading to Paradoxical Improvement of Glucose Tolerance

**DOI:** 10.3390/ijms27219968

**Published:** 2026-11-06

**Authors:** Mikyoung You, Soonkyu Chung

**Affiliations:** 1 Department of Food and Nutrition, Mokpo National University, Muan-Gun, Jeonnam-Gwangju Special Metropolitan City 58554, Republic of Korea; 2 Convergence Center for Green Anti-Aging Research, Mokpo National University, Muan-Gun, Jeonnam-Gwangju Special Metropolitan City 58554, Republic of Korea; 3 Department of Nutrition and Health Sciences, University of Nebraska, Lincoln, NE 68583, USA; soonkyuchung@umass.edu; 4 Department of Nutrition, University of Massachusetts, Amherst, MA 01003, USA

**Keywords:** IRP1, erythropoiesis, RBC, glucose disposal

## Abstract

Iron regulatory receptor 1 (IRP1) plays a key regulatory role in iron homeostasis, hypoxia sensing, and erythropoiesis. IRP1 deficiency causes polycythemia in early life, which is worsened by a low-iron diet. A high-fat (HF) diet is a prevalent metabolic insult that causes local hypoxia in adipose tissue, but little is known about the role of IRP1-mediated metabolic adjustments in preserving iron balance. In this study, we hypothesized that deletion of IRP1 fails to control HF-diet-induced hypoxia, thereby exacerbating diet-induced obesity and insulin resistance. HF feeding in IRP1-deficient mice induced a dramatic increase in EPO production, aberrant erythropoiesis, and splenomegaly through the reinstatement of the HIF2 α/EPO pathway. The deletion of IRP1 did not alter body weight gain but caused a paradoxical increase in glucose tolerance compared with WT mice. The 2-DOG uptake assay showed higher RBC-associated radioactivity in IRP1-deficient mice. RBC metabolomic analysis further demonstrated that IRP1 deficiency is associated with elevated metabolite levels generated by glycolysis and the pentose phosphate pathway. Collectively, our work demonstrates that IRP1 plays a critical role in preventing abnormal RBC formation under HF diet-mediated hypoxic conditions. Our work also provides an unexpected insight into RBC metabolism as a novel target against insulin resistance.

## 1. Introduction

Iron is an important micronutrient in our body and plays a pivotal role in oxygen transport, energy production, DNA synthesis, and cellular respiration [1,2]. Iron dysregulation, either deficiency or overload, is associated with the pathogenesis of diverse disease conditions ranging from anemia to cardiovascular, pulmonary, and metabolic diseases. The cellular iron pool is tightly regulated by iron regulatory proteins (IRPs) in post-transcriptional regulation [3]. Iron regulatory proteins (IRPs) bind to mRNA containing the iron response elements (IREs) at the 5′ or 3′ untranslated region (UTR), thereby inhibiting mRNA translation or increasing mRNA stability [1,3]. Recently, it was discovered that IRPs play a fundamental role in neutrophil development and differentiation in the bone marrow [4] and in neurodegenerative disease [5]. IRP1 is a dual-functional protein with a 4Fe-4S cluster that acts either as a cytosolic aconitase (holo-IRP1) or as an IRE-binding protein (apo-IRP1) [6,7,8,9]. Cytosolic aconitase implants a 4Fe-4S cluster in the presence of high iron levels, conferring enzymatic activity while losing IRE-binding activity [6,7,8,10]. Under iron-deficient conditions, aconitase loses the 4Fe-4S cluster and functions as an IRE-binding protein.

It has been proposed that IRP1 is an initiator of polycythemia [11,12,13,14]. Under normal conditions, IRP1 is present in the apo-form, which binds to the IRE region of hypoxia-inducible factor 2 alpha (HIF2α), repressing its translation [15]. However, lack of IRP1 prevents binding to Hif2α mRNA, resulting in increased HIF2α synthesis [16]. It can accelerate erythropoietin (EPO) production, resulting in erythropoiesis/polycythemia. Intriguingly, HIF2α-mediated erythropoiesis in the IRP1^−/−^ mice occurs restricted to early life, juvenile animals, and recovers at a normal level after 10 weeks old [12,14].

With the progress of the study on obesity, researchers discovered that adipocyte hypotrophy causes local hypoxia in adipose tissues owing to an insufficient circulatory network [17,18,19,20,21]. The local hypoxia in adipose tissue is associated with dysregulated adipokines [18,20], glucose intolerance [17,21], and inflammation [19,20,21], suggesting that local hypoxia is a trigger for obesity. Recent insights in the field of hypoxia have focused on non-alcoholic fatty liver disease (NAFLD) in humans [22] and mouse models [22,23,24,25]. Oxygen therapy attenuated hypoxia and inhibited HIF-2α signaling in the liver, resulting in diminished NAFLD [26]. Given the importance of hypoxia during obesity, nothing is known about how lack of IRP1 worsens the local hypoxia during diet-induced obesity (DIO). Here, we hypothesized that loss of IRP1 aggravates DIO and insulin resistance by failing to regulate HF-diet-induced hypoxia.

## 2. Results

### 2.1. Loss of Irp1 Kept Erythropoiesis via Hif2α/Epo Signaling upon High Fat Feeding

**Figure 1:**
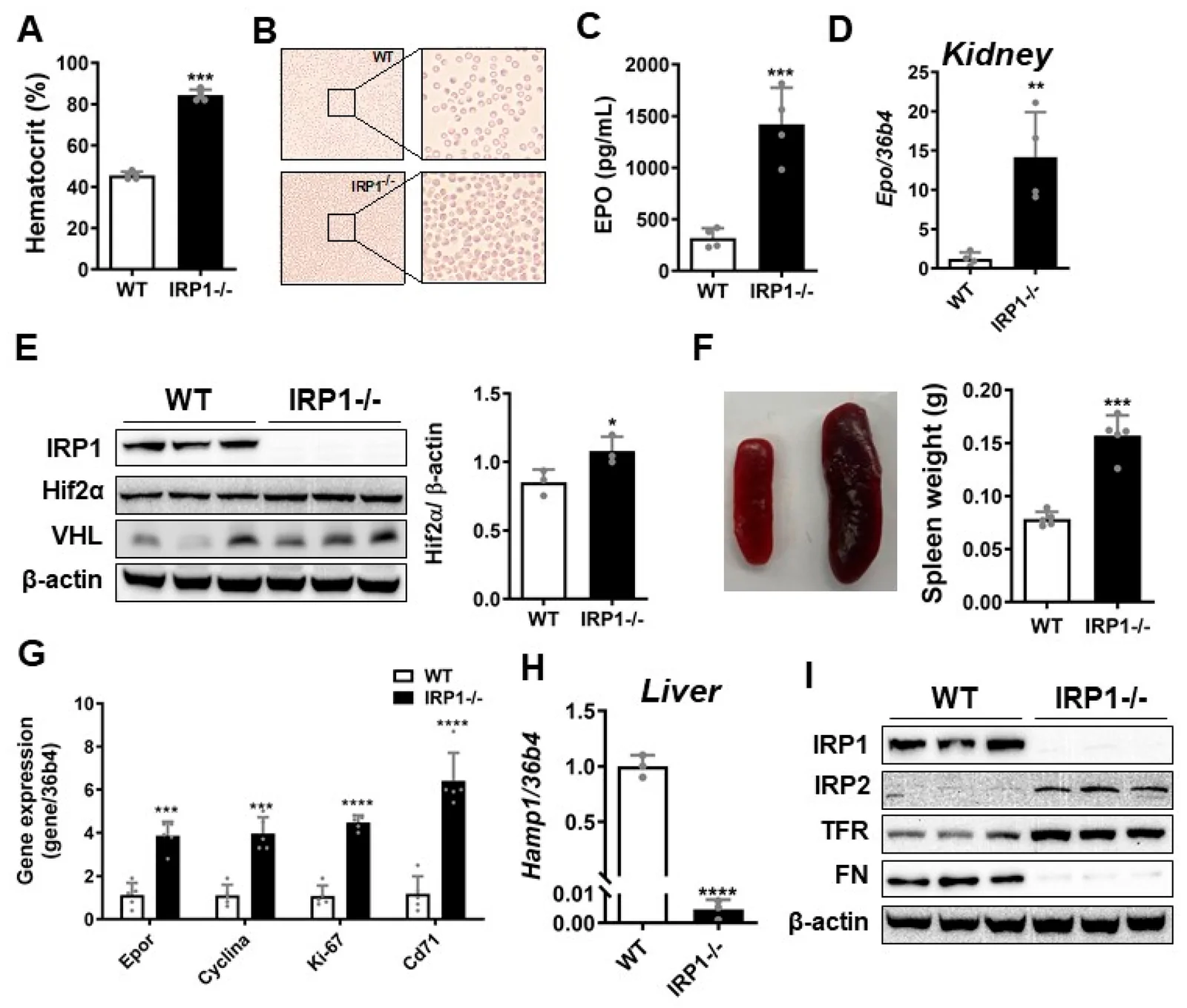
Hematological, renal, splenic, and hepatic characteristics of HFD-fed WT and IRP1 knockout mice. (**A**) Hematocrit levels. (**B**) Wright–Giemsa staining of peripheral blood (40× magnification). (**C**) Plasma EPO levels. (**D**) mRNA expression level of *Epo* in the kidney. (**E**) Western blot analysis of IRP1, Hif2α, and VHL in the kidney (**left**) and Hif2α relative intensities to β-actin quantified by Image J (**right**). (**F**) Gross images of the spleen (**left**) and spleen weight (**right**). (**G**) mRNA expression levels of erythroid differentiation markers, including Epor, CyclinA, Ki-67, and Cd71, in the spleen. (**H**) mRNA expression level of *Hamp1* in the liver. (**I**) Western blot analysis of IRP1, IRP2, TFR1, and FN in the liver. All data are shown as mean ± SD. * *p* < 0.05; ** *p* < 0.01; *** *p* < 0.001; **** *p* < 0.0001 by Student’s *t*-test.

IRP1 deficiency results in the upregulation of erythropoietin (EPO) production in the kidney caused by translational activation of Hif2α [12]. Based on this literature, we examined erythropoiesis in the HF-fed IRP1^−/−^ mice. The hematocrit levels were two-fold higher in the IRP1 KO mice (Figure 1A). Analysis of peripheral blood smears by Wright–Giemsa staining showed pathologically increased numbers of reticulocytes in IRP1^−/−^ mice compared to WT mice (Figure 1B). Subsequently, serum EPO level and *Epo* mRNA expression in the kidney were higher in the IRP1^−/−^ mice by three- and 15-fold, respectively (Figure 1C,D). Consistent with the results of the EPO levels, the Hif2α and VHL protein levels in the kidney were increased in the IRP1^−/−^ mice (Figure 1E). Strikingly, spleen size and weight were elevated to as high as two-fold in these IRP1^−/−^ mice (Figure 1F). The expression levels of the erythropoiesis receptor (*Epor*), proliferation factors (*Cyclina* and *Ki-67*), and iron uptake receptor (*Cd71*) significantly increased in the IRP1^−/−^ mice compared to WT mice (Figure 1G). To examine iron regulation in the liver, we measured systemic and cellular iron regulators. Hepatic *Hamp1* expression, the hepcidin gene involved in systemic iron regulation, was dramatically decreased in IRP1^−/−^ mice (Figure 1H). In response to downregulation of *Hamp1* expression in the liver, IRP2 and TFR1 increased, and FNL decreased in the IRP1 KO mice (Figure 1I). These results indicate that the absence of IRP1 with HF feeding leads to extreme erythropoiesis, not only in juveniles.

### 2.2. Irp1 Deletion Does Not Alter Hf-Diet-Induced Weight Gain Despite Increased Food Intake and Decreased Physical Activity

**Figure 2:**
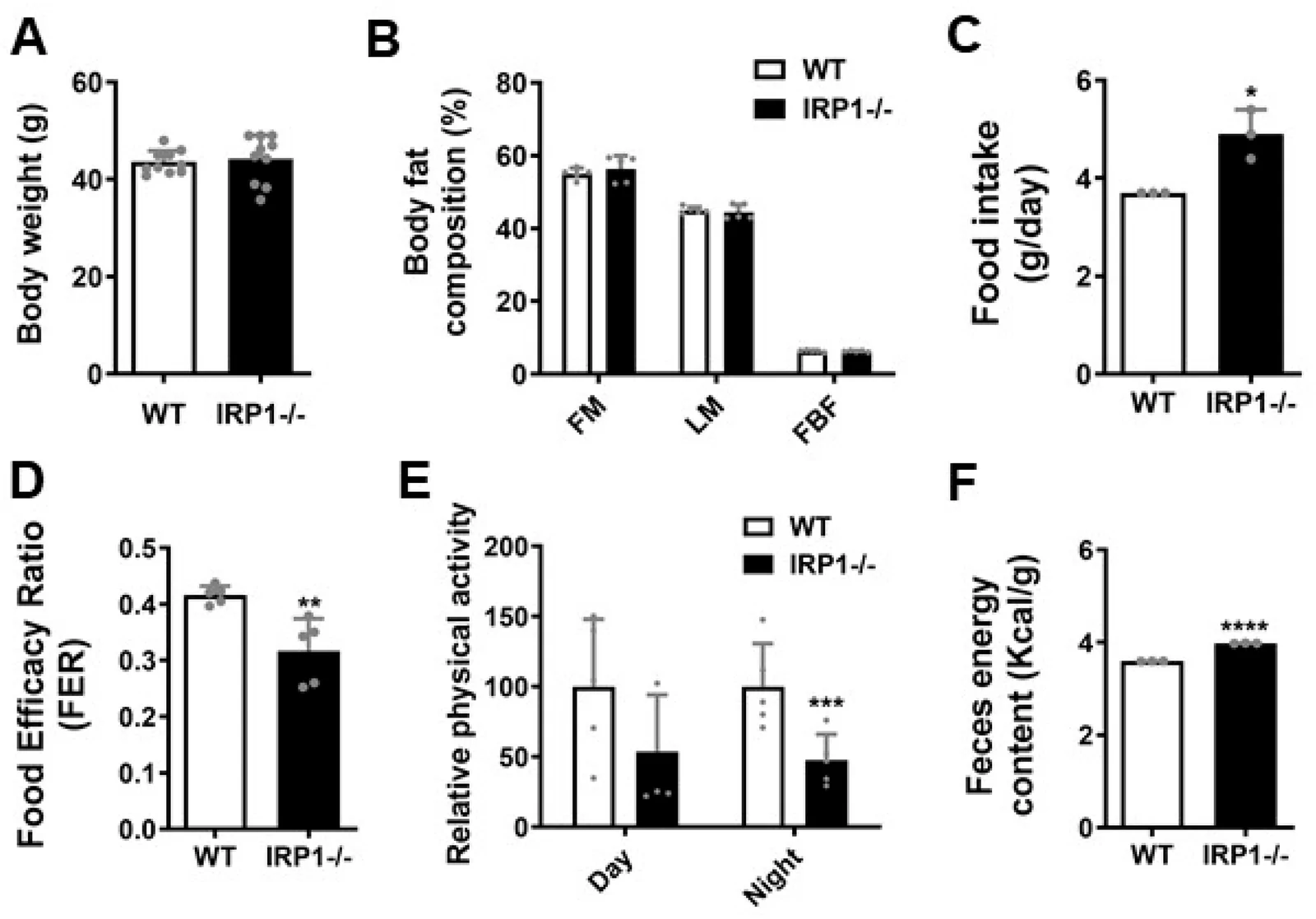
IRP1 deletion does not alter body weight gain despite increased food intake, relative activity, and fecal mass. Five-week-old wild-type and IRP1^−/−^ male mice (*n* = 10) were fed HFD (45% Kcal fat containing 0.2% cholesterol) for 14 weeks. (**A**) Final body weight. (**B**) Body fat composition (FM: fat mass, LM: lean mass, and FBF: free body fluid). (**C**) Food intake. (**D**) Food efficacy ratio (calculated by body weight gain/food intake). (**E**) Relative physical activity. (**F**) Feces energy content. All data are shown as mean ± SD. * *p* < 0.05; ** *p* < 0.01; *** *p* < 0.001; **** *p* < 0.0001 by Student’s *t*-test.

The body weight did not differ between WT and IRP1^−/−^ mice upon HF feeding (Figure 2A). Consistent with the body weight, body fat composition did not change in the IRP1 KO mice (Figure 2B). Despite no differences in body weight between WT and IRP1^−/−^ mice had significantly higher food intake (Figure 2C) with altered satiety hormones (Supplementary Figure S1). In addition, the food efficacy ratio (FER) was significantly decreased in the IRP1^−/−^ mice (Figure 2D). Next, we asked whether the IRP1 deletion alters physical activity and fecal energy, as measured by the metabolic cage and the bomb calorimeter, respectively. The relative physical activity levels were lower in the IRP1^−/−^ mice than in WT mice at nighttime (Figure 2E), whereas fecal energy content was higher in IRP1^−/−^ mice (Figure 2F), indicating that the energetic balance of IRP1^−/−^ mice should be shifted toward increased energy expenditure. Taken together, these results demonstrate that IRP1^−/−^ mice maintain stable body weight despite high food intake and low activity.

### 2.3. Irp1 Deletion Alters Glucose Homeostasis and Increases Glucose Uptake in Rbcs

**Figure 3:**
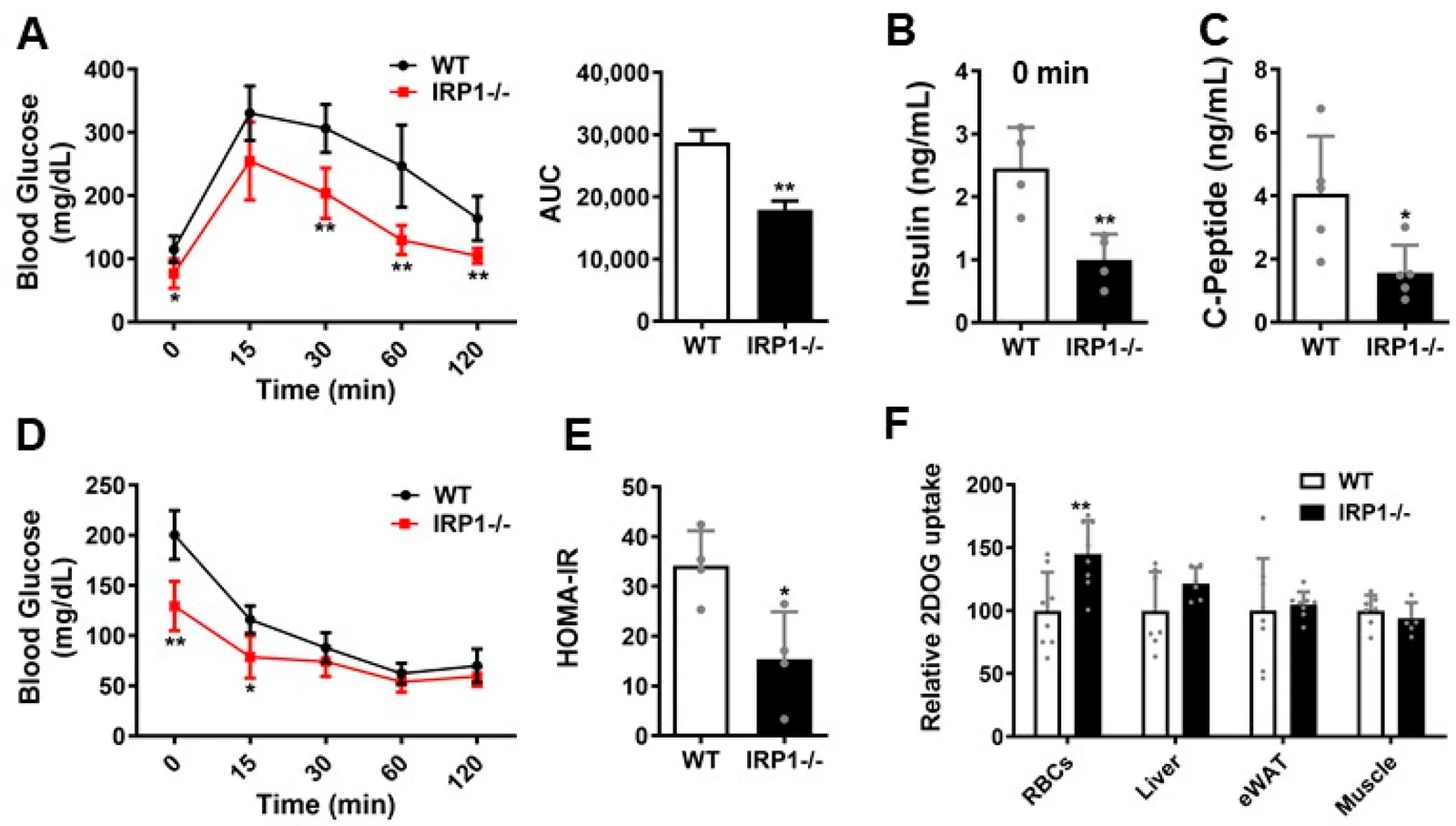
IRP1 deletion promoted glucose disposal from the circulation without improving insulin sensitivity. (**A**) Glucose tolerance test (GTT) and the area under the curve (AUC). (**B**) Fasting insulin levels. (**C**) Serum C-peptide levels. (**D**) Relative insulin tolerance test (ITT). (**E**) HOMA-IR. (**F**) Relative ^3^H-2-deoxy-d-glucose (2DOG) uptake. All data are shown as mean ± SD. * *p* < 0.05; ** *p* < 0.01 by Student’s *t*-test.

To assess whether the ability to regulate insulin sensitivity was impacted by IRP1 deletion, we first performed a glucose tolerance test (GTT). Glucose disposal was remarkably faster in IRP1^−/−^ mice than in WT mice. IRP1^−/−^ mice had an improved glucose tolerance, as confirmed by quantification of the GTT area under the curve (AUC) (Figure 3A). Levels of insulin and C-peptide (proinsulin) under fasting conditions were significantly lower in IRP1^−/−^ mice than in WT mice, indicating that IRP1^−/−^ mice regulate glucose levels without increasing insulin levels (Figure 3B,C). We next assessed the blood glucose response to exogenous insulin using an insulin tolerance test (ITT; 1 U/kg human insulin). Blood glucose declined in both genotypes, with broadly similar response profiles (Figure 3D). Importantly, HOMA-IR was lower in IRP1^−/−^ mice (Figure 3E); we therefore report this as a lower HOMA-IR rather than concluding that IRP1 deletion suppresses insulin resistance.

Next, we attempted to assess the glucose uptake involved in glucose disposal across the whole-body system, including plasma, RBCs, liver, adipose tissue, and muscle, as measured by a ^3^H-2-deoxy-D-glucose (2-DOG) assay (Figure 3F). The changes in ^3^H-2DOG uptake for various organs were shown in Figure 3F. Notably, the radioactivity concentration in RBCs was significantly increased in the IRP1^−/−^ mice, while no significant changes were observed in other tissues. Collectively, these results suggest that the increased glucose disposal in IRP1^−/−^ mice is likely due to increased glucose uptake specifically in red blood cells.

### 2.4. Energy Metabolism in Red Blood Cells Was Attenuated by Irp1 Deletion

**Figure 4:**
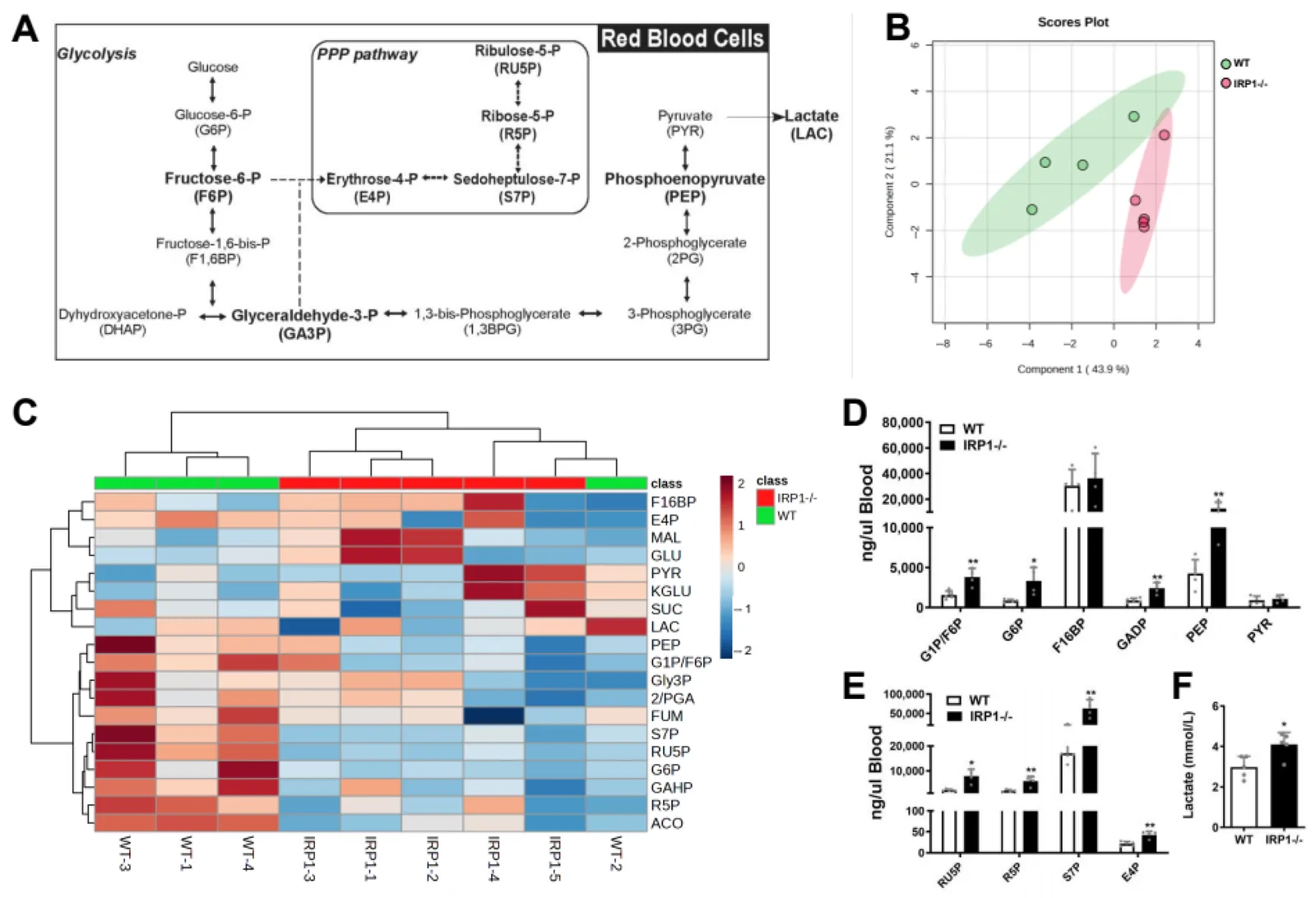
IRP1 deletion induced metabolic reprogramming of red blood cells (RBCs). (**A**) An overview of the interaction of glycolysis (solid arrows) and the pentose phosphate pathway (PPP, dashed arrows) in RBCs. Metabolites from RBCs were measured by metabolomics using UHPLC-MS. (**B**) sPLS-DA score plot showing the separation between WT and IRP1^−/−^ groups. The shaded ellipses represent the group-specific confidence regions. (**C**) Heat map analysis. (**D**) Glycolysis pathway. In each graph, the Y-axes are normalized to Hct levels. (**E**) Pentose phosphate pathway (PPP). In each graph, the Y-axes are normalized to Hct levels. (**F**) Plasma lactate levels measured by ELISA. All data are shown as mean ± SD. * *p* < 0.05; ** *p* < 0.01 by Student’s *t*-test.

To determine the potential interaction between erythropoiesis and energy metabolism in IRP1^−/−^ mice, we performed UHPLC analysis of metabolites from glycolysis and the pentose phosphate pathway (PPP) in RBCs (Figure 4A). sPLS-DA was used to evaluate discriminative differences between WT and IRP1^−/−^ mice (Figure 4B), as further illustrated by the generated heat maps (Figure 4C). Hct-adjusted levels of several glycolytic metabolites, including glucose-1-phosphate (G1P), fructose-1,6-bisphosphate (F1, 6BP), glyceraldehyde-3-phosphate (G3P), phosphoenolpyruvate (PEP), and pyruvate (PYR), were higher in IRP1^−/−^ mice (Figure 4D). Ribose-5-phosphate (R5P) and erythrose-4-phosphate (E4P), metabolites of the PP pathway, also showed higher levels in RBCs from IRP1^−/−^ mice (Figure 4E). In contrast, lactate levels in the serum, not RBCs, was significantly enhanced in IRP1^−/−^ mice (Figure 4F). These data indicated that the RBCs’ energy metabolism in IRP1^−/−^ mice was increased and that energy expenditure shifted to whole-body energy expenditure.

## 3. Discussion

IRP1 deficiency in mouse models has shown extreme erythropoiesis, driven by HIF2α signaling in early life [4,10,12,13,14]. Local hypoxia, caused by a high-fat (HF) diet, plays a pivotal role in aggravating obesity and obesity-related diseases. However, it is unknown whether the HF diet caused the hypoxia condition to persist in IRP1 KO mice, worsening their obesity or obesity-related disease. In this study, we demonstrated the impact of IRP1 deficiency on obesity and insulin resistance resulting from the HF diet. Our findings reveal that IRP1 deletion sustains erythropoiesis via activating HIF2α signaling under the HF diet condition. Interestingly, IRP1 deficiency did not affect body weight, despite the increased food intake and decreased physical activity. Moreover, the lack of IRP1 results in a decrease in plasma glucose levels, with no insulin resistance due to enhanced glucose uptake in RBCs, which occurs more rapidly than in other tissues. Increasing RBCs contributed to energy expenditure through increasing glycolysis and the pentose phosphate pathway. Our present findings provide the first novel metabolic evidence that IRP1 deletion plays a key role in RBC energy expenditure.

Previous studies have shown increased erythropoiesis in young IRP1 KO mice, although this phenotype may differ with age and iron status. Ghosh et al. [11] reported that iron deficiency increased hematocrit in IRP1^−/−^ mice, but did not observe levels near 80%. In our study, hematocrit in HF-fed IRP1^−/−^ mice reached levels comparable to those reported at 4–6 weeks of age. This difference may reflect the different experimental conditions. Our results suggest that erythropoiesis remains elevated in HF-fed IRP1^−/−^ mice, but do not show whether the HF diet reactivated this phenotype or whether it persisted from early life. HIF-2α is a central regulator of hypoxia-induced erythropoiesis, and mutations in HIF-2α genes, hypoxia-inducible factor prolyl hydroxylase 1 (PHD1), or VHL have been associated with abnormal erythropoietic responses [27]. Hif2α is primarily regulated by iron metabolism and erythropoietin (EPO) synthesis, whereas Hif1α is more closely associated with anaerobic glycolysis [28]. Under normoxic conditions, prolyl hydroxylation targets HIF2α for recognition by the VHL E3 ubiquitin ligase, leading to ubiquitination and proteasomal degradation. Conversely, hypoxia or impaired VHL activity stabilizes HIF2α and promotes transcription of its target genes [29,30]. The importance of this pathway is further demonstrated by *Vhl^R220W^* mutant mice, in which impaired VHL function causes dysregulated erythropoiesis and severe pathological abnormalities [11]. Thus, the increase in VHL protein observed in HF-fed IRP1^−/−^ mice may impair HIF2α degradation, resulting in persistent HIF2α activation and continued stimulation of erythropoiesis into adulthood (Figure 1).

Increasing evidence suggests that hypoxia can improve glucose metabolism in individuals with obesity [31,32]. In healthy obese men, ten nights of moderate hypoxia improved whole-body insulin sensitivity, potentially by increasing skeletal-muscle glucose uptake through insulin-independent mechanisms [31]. However, the metabolic effects of VHL signaling appear to be tissue-specific. Pancreatic β-cell-specific Vhl deletion impaired glucose tolerance, β-cell function, and β-cell mass, partly by reducing GLUT2 expression [33], whereas osteoblast-specific Vhl deletion lowered fasting blood glucose, improved glucose tolerance, enhanced glycolysis, and produced a lean phenotype [34]. These findings suggest that persistent hypoxia in HF-fed IRP1^−/−^ mice contributes to reduced glucose excursion and HOMA-IR via tissue-specific metabolic adaptations rather than a generalized enhancement of insulin action (Figure 2).

Guo et al. [35] reported comparable metabolic benefits in obese adults, including weight loss and changes in fasting glucose levels. However, the metabolic consequences of hypoxia may depend on both the underlying metabolic state and the hypoxic exposure protocol. Chronic intermittent hypoxia has been shown to induce insulin resistance and non-alcoholic fatty liver disease in obese mice, whereas lean mice were less susceptible to these adverse effects [36,37,38]. In contrast, IRP1^−/−^ mice exhibited reduced hepatic lipid accumulation compared with wild-type mice (Supplementary Figure S2), suggesting that the metabolic phenotype associated with IRP1 deficiency may differ from the pathological response to intermittent hypoxia in obesity.

Recent work further indicates that hypoxia improves glucose tolerance, in part, by increasing erythrocyte mass and glucose utilization [38]. Hypoxia-induced erythrocytes exhibit approximately three-fold greater glucose uptake and a two-fold increase in GLUT1 abundance, thereby enhancing glycolysis and 2,3-diphosphoglycerate production. In our study, relative 2-deoxyglucose uptake was higher in the RBC fraction of 5-week-old IRP1^−/−^ mice, while no comparable increase was observed in the other tissues examined (Figure 3). Metabolomic profiling of RBCs also revealed changes in metabolites associated with glycolysis and the pentose phosphate pathway (Figure 4), consistent with altered RBC glucose metabolism. Together, these findings suggest that increased erythrocyte mass and glucose utilization may provide an additional glucose-utilizing compartment, thereby contributing to systemic glucose handling. However, although RBCs were washed twice with PBS before metabolomic analysis, residual plasma contamination cannot be completely ruled out. In addition, IRP1 aconitase activity was not directly assessed in mature RBCs; therefore, the observed metabolite changes cannot be specifically attributed to altered IRP1 aconitase function. The energetic demands of erythropoiesis and RBC metabolism may also explain the observation that body weight did not increase despite greater food intake and reduced physical activity.

One thing is that hepatic hepcidin downregulation is a major regulator of systemic iron homeostasis and can increase intestinal iron absorption and cellular iron uptake through divalent metal transporter 1 (DMT1) and transferrin receptor 1 (TfR1), respectively. Excess iron may accumulate in organs such as the pancreas, where it can promote oxidative injury, inflammation, and fibrosis [39]. However, pancreatic iron staining did not differ between wild-type and IRP1^−/−^ mice (Supplementary Figure S3), suggesting that IRP1 deficiency does not cause overt pancreatic iron deposition under the conditions examined. This finding raises the possibility that increased iron availability may instead support erythropoiesis and red blood cell production. Nevertheless, direct assessment of bone marrow iron utilization, erythroid differentiation, and erythrocyte turnover will be necessary to determine whether IRP1 deficiency promotes preferential allocation of iron to erythropoiesis.

In our liver samples, IRP1 deficiency was associated with increased TFR1 and reduced ferritin. This pattern differs from the predominant pattern reported by Gu et al. [40], although they also observed increased TFR1 and reduced ferritin in two of nine IRP1^−/−^ liver samples. Differences in genetic background and experimental conditions, including age and diet, may contribute to the variation between studies. However, these factors were not directly tested in our study, and their contribution to the differing observations remains uncertain.

Several limitations should be considered when interpreting our findings. First, energy expenditure was not directly measured; therefore, the possibility that increased erythropoiesis or RBC glucose metabolism contributed to whole-body energy balance remains a hypothesis and should be tested using indirect calorimetry and erythrocyte-turnover studies. Second, increased RBC glucose uptake was observed in 5-week-old IRP1^−/−^ mice, and whether this finding also occurs in adult HF-fed mice remains unknown. Because RBC GLUT1 abundance was not measured, its contribution to the observed glucose uptake also requires further study. In addition, direct assessment of bone marrow iron utilization, erythroid differentiation, and RBC turnover would help clarify how IRP1 deficiency affects erythropoiesis. The functional status of VHL and HIF-2α turnover was not established, and we did not determine whether IRP2 or other iron-regulatory pathways compensate for IRP1 loss. Finally, although genetic background, age, and diet may help explain differences between our hepatic findings and those of previous studies, their individual contributions remain uncertain.

In conclusion, IRP1 deficiency increases energy expenditure despite greater food intake and reduced physical activity. IRP1 KO mice also exhibited enhanced glucose disposal and reduced insulin resistance, accompanied by sustained erythropoiesis and increased glucose utilization by red blood cells. These findings suggest that IRP1 loss induces metabolic reprogramming that expands the erythroid compartment and enhances its contribution to whole-body energy metabolism. Although the underlying mechanisms require further investigation, our results identify IRP1 as a potential regulator of erythrocyte-associated energy expenditure and provide new insight into the role of red blood cells in systemic metabolic homeostasis.

## 4. Materials and Methods

### 4.1. Animals

All protocols and procedures were approved by the Institutional Animal Care and Use Committee of the University of Nebraska-Lincoln. The C57BL/6 male mice were obtained from Jackson Laboratory (Bar Harbor, ME, USA). Animals were maintained under controlled environmental conditions (20–24 °C, 40–60% relative humidity, and a 12 h light/12 h dark cycle), with water available ad libitum. Homozygous IRP1^−/−^ mice on a C57BL/6 background were kindly provided by Dr. Tracey Rouault (Section on Human Iron Metabolism, National Institute of Child Health and Human Development, National Institutes of Health, Bethesda, MD, USA). The original paper describing these mice is cited in [11]. IRP1^−/−^ mice were derived from homozygous breeding pairs in our colony. Offspring were weaned at 3 weeks of age and were genotyped by amplifying 350 bp (wild-type) and 450 bp (KO) fragments from the IRP1 gene, using the primers PGK-1: AGCTCATTCCTCCACTCATG, IRP1Rev: GCATGCATCCATTGTCTCTG, and IRP1Rev: ACAGACACAGATGCCAGAGG (Supplementary Figure S4). To identify general characteristics of IRP1^−/−^ mice, mice were fed a normal chow diet, starting at weaning for 7 weeks. Body weight, organ weight, serum EPO level, Hif2a protein expression in the kidney, and Hamp1 mRNA expression were measured after necropsy.

For the high-fat diet (HFD) feeding study, five-week-old mice were fed an HFD providing 45% of total calories from fat and containing 0.2% cholesterol (Table S1) for 14 weeks. The diet was prepared in-house based on the AIN-93M formulation [41]. The manufacturers and catalog numbers of the individual ingredients are listed in Table S1. Body weight and food intake were monitored weekly. Food efficacy ratios were calculated as body weight gain/food intake. At the time of necropsy, plasma, red blood cells (RBCs), liver, spleen, kidney, and pancreas were collected and kept at −80 °C until analysis.

### 4.2. Measurement of Body Composition, Relative Activity, and Feces Energy Content

Body composition was measured using a Minispec Lf50 Body Composition Analyzer (Bruker Corporation, Billerica, MA, USA), and fat mass, lean mass, and free body fluid were analyzed using the Minispec software (*n* = 5, Bruker Corporation, Billerica, MA, USA (https://www.bruker.com/en/products-and-solutions/mr/nmr-epr-td-nmr-industrial-solutions/minispec-mq-one.html?f_link_type=f_linkinlinenote&flow_extra=eyJpbmxpbmVfZGlzcGxheV9wb3NpdGlvbiI6MCwiZG9jX3Bvc2l0aW9uIjowLCJkb2NfaWQiOiIwNjM3Nzk3NGNlOTA0MzY3LTM2MWI2M2NkNTJiODBhZDAifQ%3D%3D, accessed on 31 October 2026)). Relative activity was performed using a metabolic cage (TSE systems, Chesterfield, MO, USA) as described previously [42]. Briefly, mice were placed into an individual cage for 4 days (*n* = 5). Data were normalized by the activity of WT mice at night as active time. To measure feces energy content, the mice were placed into a clean cage for three days (*n* = 5). After 3 days, feces were collected, dried, homogenized, and measured for energy content using a bomb calorimeter (Parr 6400 Calorimeter, Parr Instrument Company, Moline, IL, USA).

### 4.3. Glucose and Insulin Tolerance Test

Glucose tolerance test (GTT) and insulin tolerance test (ITT) were measured as we described previously [42,43]. Briefly, mice were fasted overnight before each test and injected with 10% D-glucose solution (1 g/kg body weight) for GTT and 1U of insulin for ITT (*n* = 10). Glucose disposal from the circulation was measured using a glucometer (Bayer Contour^®^, Ascensia Diabetes Care, Parsippany, NJ, USA) at a specified time point. Fasting blood was collected, and insulin levels were measured using an Ultra-Sensitive Mouse Insulin ELISA kit (Crystal Chem, Elk Grove Village, IL, USA) according to the manufacturer’s protocol. For the assessment of insulin resistance, the HOMA-IR (homeostasis model assessment of insulin resistance) index was calculated as fasting plasma glucose × fasting plasma insulin/22.5.

### 4.4. ^3^H-2DOG Uptake Assay

To measure 2-Deoxy d-glucose (2DOG) uptake in glucose-responsible organs, we used 5-week-old IRP1 KO mice due to a similar hematocrit level to that of HF-fed IRP1 KO mice (80% vs. 85%). Mice were placed into an individual cage and intraperitoneally injected with 10 uCi of ^3^H-2DOG for 45 min. Following Sokoloff et al. [44], a 45 min injection with 2DOG reaches a maximum level in tissues and a very low level in plasma. After 45 min, mice were sacrificed, and red blood cells, liver, epididymal adipose tissue, and muscle were collected, and radioactive tracers were measured by liquid scintillation counting as described previously [45]. These results were normalized by organ weight.

### 4.5. Blood Analysis

On the day of necropsy, blood was collected and treated with a DPPIV inhibitor (Millipore, Burlington, MA, USA) and a protease inhibitor cocktail (Sigma, St Louis, MO, USA) for the satiety hormone using a Mouse Metabolic Magnetic Bead Panel (Milliplex, Millipore). Leptin and adiponectin levels were measured using ELISA kits according to the manufacturer’s protocol (R&D Systems, Minneapolis, MN, USA and Crystal Chem, respectively). Plasma EPO levels were detected using a colorimetric assay (R&D Systems). To test the blood smear, whole blood was put onto the slide and was stained with Wright–Giemsa stain solution following the manufacturer’s protocol (Thermo Fisher Scientific, Waltham, MA, USA). After staining, the slide was examined under a microscope (EVOS Cell Imaging System, Thermo Fisher Scientific).

### 4.6. Western Blotting Analysis

The protein was extracted using an RIPA buffer containing inhibitors of protease and phosphatase (Sigma). Proteins were fractionated using 10% SDS-PAGE, transferred to PVDF membranes, and incubated with the relevant antibodies conjugated with horseradish peroxidase (HRP). All antibodies used for experiments are summarized in Table S2. Chemiluminescent signals were developed using Western Lightning Plus-ECL (PerkinElmer, Waltham, MA, USA) and detected using an ODYSSEY FC Imaging System (LI-COR, Lincoln, NE, USA).

### 4.7. Qpcr

Total RNA from tissues was isolated using Trizol^®^ reagent (Invitrogen, Carlsbad, CA, USA) and purified. Gene expression was determined by real-time qPCR (QuantStudio 6 Flex, Applied Biosystems, Foster City, CA, USA) using SYBR green (Thermo Fisher Scientific), and relative gene expression was determined based on the 2^−ΔΔCT^ method with normalization of the raw data to *36b4* (primer sequences in Table S3).

### 4.8. H&E and Prussian Blue Staining

The liver and pancreatic tissues were fixed in 10% buffered formalin, embedded in paraffin, and cut into 5 µm sections. The liver was stained with hematoxylin and eosin (H&E). To measure pancreatitis by iron overload, pancreas sections were stained with Perl’s Prussian blue staining as we described previously [43].

### 4.9. Metabolomics Analysis

To measure energy expenditure in red blood cells, whole blood was collected from submental veins after fasting. Blood was centrifuged and separated into serum and red blood cells. After the RBC collection, it was resuspended in PBS twice and then washed. Then 100 uL of RBC was mixed with 600 uL of extraction buffer (water: chloroform: methanol = 3:5:12 *v*/*v*) and PIPES [piperazine-N,N′-bis (2-ethanesulfonic acid)] as an internal standard and was centrifuged at 16,000× *g* for 15 min. The supernatant was collected and centrifuged again. The supernatant was pooled, and 300 uL chloroform and 450 uL of chilled water were added. After centrifugation, the upper layer was transferred to a new tube and dried down using a speed-vac. The pellets were re-dissolved in 100 uL of 80% acetonitrile. For LC separation, Poroshell 120 (Agilent, Santa Clara, CA, USA, 2.7 um, 150 × 2.1 mm) was used, flowing at 0.5 mL/min. The gradient of the mobile phases A (20 mM ammonium acetate, pH 9.8, 5% CAN) and B (100% acetonitrile) was as follows: 85% B for 1 min, to 40% B in 9 min, holding at 40% B for 2 min, then back to 85% B in 0.5 min. The LC system was interfaced with a Sciex QTRAP 6500+ mass spectrometer (SCIEX, Marlborough, MA, USA) equipped with a TurbolonSpray (TIS) electrospray ion source. Analyst software (version 1.6.3) was used to control sample acquisition and data analysis. The QTRAP 6500+ mass spectrometer was tuned and calibrated according to the manufacturer’s recommendations. All the metabolites were detected using MRM transitions that were previously optimized using standards. The instrument was set up to acquire data in positive/negative mode switching. For quantification, an external standard curve was prepared using a series of standard samples containing different concentrations of metabolites and a fixed concentration of the internal standard. Sparse partial least squares discriminant analysis (sPLS-DA) and heat map analysis were performed using MetaboAnalyst (version 6.0, https://www.metaboanalyst.ca/). Metabolite abundances were quantified in RBC samples (ng/mL). For Figure 4D,E, values were converted to whole-blood equivalents by multiplying each measurement by the corresponding hematocrit fraction; unadjusted values are shown in Figure 4C.

### 4.10. Statistical Analysis

All data were analyzed using one-way ANOVA, followed by Tukey’s multiple comparison tests or Student’s *t*-test; * *p* < 0.05 and ** *p* < 0.01. For the analysis of the statistical significance in GTT, ITT, area under the curve, and metabolomics, data were calculated from individual animals, followed by analysis using one-way ANOVA. All analyses were performed using GraphPad Prism (Version 6.02).

## Data Availability

The original contributions presented in this study are included in the article/Supplementary Data. Further inquiries can be directed to the corresponding authors.

## Supplementary Materials

The following supporting information can be downloaded at: https://www.mdpi.com/article/10.3390/ijms27219968/s1.
