# Supplementary material for: High-Fat Diet Provokes Aberrant Erythropoiesis in IRP1-Deleted Mice, Leading to Paradoxical Improvement of Glucose Tolerance
Source: Int J Mol Sci. 2026 Nov 6;27(21):9968. doi: 10.3390/ijms27219968 (PMC13764134; doi:10.3390/ijms27219968)
Supplement: Supplementary file 1 [file ijms-27-09968-s001.zip › ijms-4651002-supplementary.pdf]

Supplemental Information

High fat diet provokes aberrant erythropoiesis in IRP1-deleted mice leading to paradoxical improvement of glucose tolerance

Mikyoung You[1,2,§], and Soonkyu Chung[3]

[1]Department of Food and Nutrition, Mokpo National University, Muan-gun, Jeonnam-Gwangju Special Metropolitan City, 58554, Republic of Korea.

[2]Convergence Center for Green Anti-Aging Research, Mokpo National University, Muan-gun, Jeonnam-Gwangju Special Metropolitan City, 58554, Republic of Korea

[3]Department of Nutrition, University of Massachusetts, Amherst, MA, 01003, USA

Inventory of Supplemental Information

Supplemental Figures

Figure S1. Levels of Appetite-Regulating Hormones and Metabolic Adipokines. (A) Ghrelin. (B) Glucose-dependent insulinotropic polypeptide (GIP). (C) Pancreatic polypeptide (PP). (D) Peptide YY (PYY). (E) Amylin. (F) Resistin. (G) Leptin. (H) Adiponectin. All data are shown as mean ± SD. * $P< 0.05$; ** $P< 0.01$ by Student's t-test.

Figure S2. H&E staining of liver.

Figure S3. H&E staining and Prussian blue staining of pancreas.

Figure S4. Genotyping results of IRP1 KO mouse

Supplemental Tables

Table S1. Diet composition

Table S2. List of primary antibodies

Table S3. Primer Sequences for qPCR

Figure S1. Levels of Appetite-Regulating Hormones and Metabolic Adipokines. (A) Ghrelin. (B) Glucose-dependent insulinotropic polypeptide (GIP). (C) Pancreatic polypeptide (PP). (D) Peptide YY (PYY). (E) Amylin. (F) Resistin. (G) Leptin. (H) Adiponectin. All data are shown as mean ± SD. * P< 0.05; ** P< 0.01 by Student's t-test

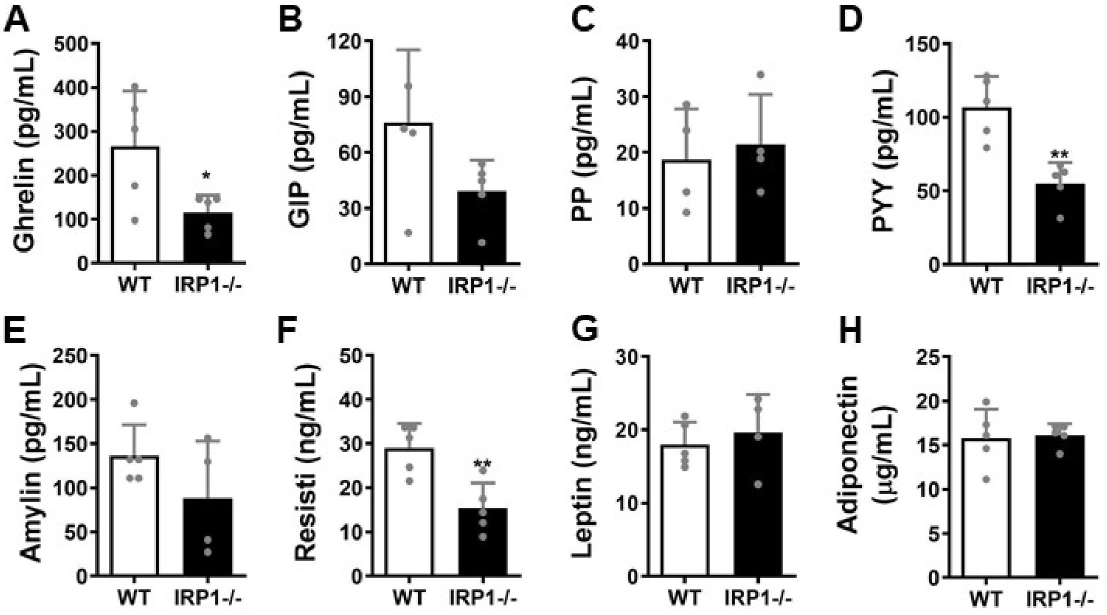

.

Figure S2. H&E staining of liver.

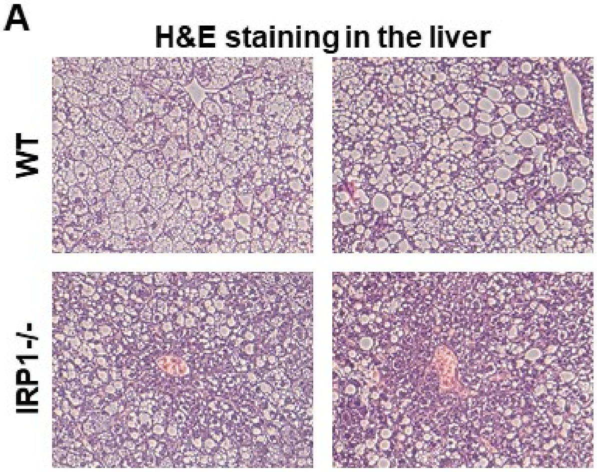

Figure S3. H&E staining and Prussian blue staining of pancreas.

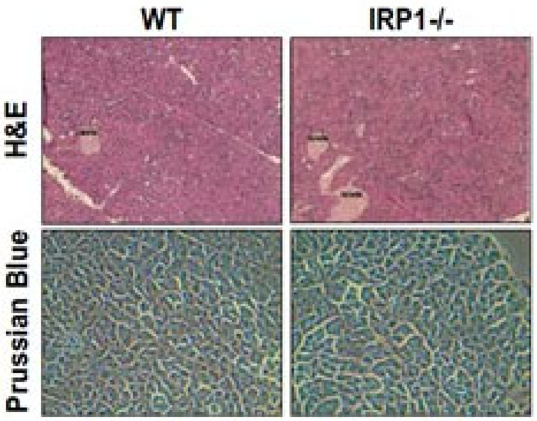

Figure S4. Genotyping results of IRP1 KO mouse

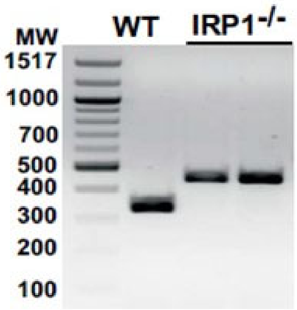

TableS1. Diet composition of HF diet

**Ingredients (g)**		Catalog Number	Company
**Casein**	235	#400600	Dyets Inc
**L-cystine**	5.5	#401340	Dyets Inc
**Corn starch**	212	#401200	Dyets Inc
**Maltodextrin**	100	#402851	Dyets Inc
**Sucrose**	100	#404400	Dyets Inc
**Lard**	220	-	Morrell Snow Cap
**Soybean oil**	20	#404360	Dyets Inc
**Cholesterol**	2	#400650	Dyets Inc
**Cellulose**	50	#401850	Dyets Inc
**Mineral mix**	35	#210050	Dyets Inc
**Calcium carbonate**	30	#400400	Dyets Inc
**Vitamin mix**	10	#300000	Dyets Inc
**Choline bitartrate**	2.5	#400750	Dyets Inc
**% calories from Fat**	45.3		
**% calories from Carbohydrate**	34.5		
**% calories from Protein**	20.2		
**Kcal/g diet**	4.67		

Table S2. List of primary antibodies

Antibody	Host	Dilution	Company	Catalog no.
IRP1	Rabbit	1:1000	Abcam	Ab236773
IRP2	Rabbit	1:1000	Thermo Fisher	Pa5-86058
TFR	Mouse	1:1000	Thermo Fisher	136800
FN	Rabbit	1:1000	Abcam	Ab69090
Hif2α	Rabbit	1:1000	Novus Biologicals	Nb100122
VHL	Mouse	1:1000	Santa Cruz Biotechnology	Sc-135657
β-actin	Rabbit	1:1000	Cell Signaling	4967

Table S3. Primer sequences for qPCR

Primer	Primer sequence	Size
Epor	F: 5'- GTCTGACTTGGCCTCAAAGC -3'<br>R: 5'- GTGAGGTGGAGTGGGAGGTA -3'	133
Cyclina	F: 5'- CTGGTTGAGGTGGGAGAAGA -3'<br>R: 5'- ATAGCAGCCGTGCCTACAAG -3'	131
Ki-67	F: 5'- ATCATTGACCGCTCCTTTAGGT -3'<br>R: 5'- GCTCGCCTTGATGGTTCCT -3'	104
Cd71	F: 5'- AGCCAGATCAGCATTCTCTAAC -3'<br>R: 5'- TCTGCAGCCAGTTTCATCTC -3'	117
Hamp1	F: 5'- CAACTTCCCCATCTGCATCT -3'<br>R: 5'- GCAGGGGTGTAGAGAGGTCA -3'	108
36b4	F: 5'- GGATCTGCTGCATCTGCTTG -3'<br>R: 5'- GGCGACCTGGAAGTCCAACT -3'	113
